# Hypertensive women with dyspnea exhibit an unfavorable central blood pressure response to exercise

**DOI:** 10.1038/s41440-025-02171-6

**Published:** 2025-03-10

**Authors:** Paulina Skalska, Małgorzata Kurpaska, Małgorzata Banak, Paweł Krzesiński

**Affiliations:** Department of Cardiology and Internal Medicine, Military Institute of Medicine National Research Institute, Warsaw, Poland

**Keywords:** Dyspnea, Exercise tolerance, Female, Hypertension, Applanation tonometry

## Abstract

Limited exercise tolerance and dyspnea in patients with uncomplicated hypertension may pose a diagnostic challenge, particularly when blood pressure is normal and assessment results do not support a diagnosis of heart failure. The purpose of this study was to assess the differences in central blood pressure (cBP) response to exercise between females with hypertension and good exercise tolerance (non-dyspneic females, nDFs; *n* = 27) and those with dyspnea on exertion (dyspneic females, DFs; *n* = 25). We also investigated the relations of cBP and its dynamics with peak oxygen consumption (peak VO_2_) and peak heart rate (peak HR) assessed by cardiopulmonary exercise test (CPET) and peak cardiac output (peak CO) assessed by impedance cardiography. Fifty-two females (mean age 54.5 ± 8.2 years) underwent applanation tonometry during CPET to assess the augmentation index (AIx), cBP, and central pulse pressure (cPP) before exercise (REST), at minute 3-rd of exercise (Ex), and at minutes: 1-st (R1) and 4-th of post-exercise rest (R2). In comparison with nDFs, DFs showed significantly higher cPP_Ex, AIx_Ex, and AIx_R1. The two subgroups showed no differences in cPP or Alx values either before exercise or at R2. In comparison with nDFs, the DFs had a less pronounced change in AIx values during post-exercise rest. There were negative correlations between peak HR and: AIx_R1, AIx_R2, change in AIx (R1–R2), between peak VO_2_ and: AIx_R1, AIx_R1–R2; between peak CO and: AIx_R1, AIx_R2, AIx_R1–R2. DFs presented a different cBP response to exercise than nDFs. Assessing cBP via applanation tonometry may prove useful in identifying hemodynamic abnormalities associated with limited exercise tolerance.

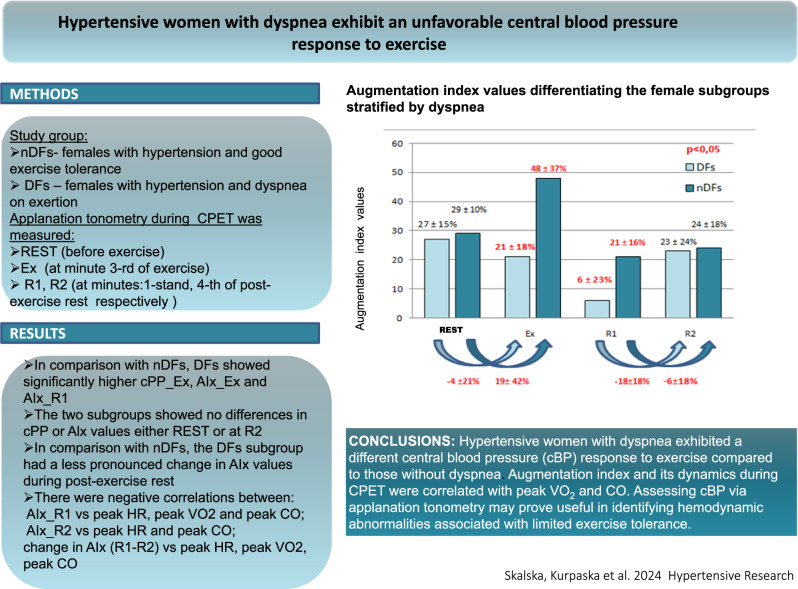

## Introduction

Dyspnea and limited exercise tolerance pose a considerable clinical and diagnostic problem when blood pressure is normal and other assessments do not support a diagnosis of heart failure. Out of all patients with hypertension (HTN) it is the women, rather than men, who report impaired exercise tolerance [1], which may be due to sex-related differences in the arterial response to exercise, which is consistent with earlier reports of sex-related differences in arterial stiffness and central blood pressure (cBP) at rest [2, 3].

Peak stroke volume (SV) and the associated cardiac output (CO) result from interactions between the heart and blood vessels [4], and depend on preload, myocardial contractility, and afterload. Moreover, cBP parameters, such as the augmentation index (AIx) and central pulse pressure (cPP), have a prognostic value and strongly correlate with left ventricular diastolic dysfunction and cardiovascular events [5, 6]. However, assessing cBP at rest may be insufficient, since the arterial response to exercise is independent from either cBP or arterial stiffness assessed at rest [7]. During exercise, there is a relative increase in peripheral blood pressure in comparison with the increase in central systolic blood pressure (cSBP) [8]. An increase in the heart rate (HR) and mean blood pressure [9] and release of paracrine hormones in vessel walls during exercise may affect vascular wall properties [10] and considerably modify cBP during exercise [7]. Intense physical exercise may substantially alter arterial stiffness, and thus reveal vascular abnormalities that are impercaptible at rest [11, 12]. Central BP during exercise may help predict future cardiovascular events better than brachial (i.e., peripheral) blood pressure does, since the former more accurately reflects left ventricular afterload [13]. Recent reports have emphasized a growing significance of large artery stiffness in heart failure development and prognosis [14].

One of our earlier studies showed that, among patients with well controlled HTN [1], women with dyspnea had normal results of routine assessments, including echocardiography and ergospirometry, and normal blood pressure; however, they showed significant abnormalities in their hemodynamic profiles assessed via impedance cardiography (ICG) during exercise. Conversely, women without dyspnea were characterized by slight changes in CO recorded between rest and peak physical exercise and lower peak SV values.

In light of the above, we posed a hypothesis that applanation tonometry assessment of cBP may provide additional value in assessing hemodynamic parameters associated with dyspnea on exertion.

## Purpose

The purpose of this study was to assess differences in the cBP response to exercise between hypertensive females with or without exercise dyspnea. We also aimed to investigate the relations of cBP and its dynamics with CPET results and hemodynamic parameters assessed (ICG).

## Methods

The study was conducted in accordance with principles of Good Clinical Practice and Declaration of Helsinki, and it was approved by the local Ethics Committee (approval No. 14/WIM/2014). All subjects had given their written informed consent. The study was registered at the Clinical-Trials.gov website (NCT02634866). This paper present the results of secondary analysis of the study.

The study group comprised female patients, aged 40–75 years, with HTN and either good exercise tolerance (non-dyspneic females, nDFs; *n* = 27) or dyspnea on exertion (dyspneic females, DFs; *n* = 25). Dyspnea on exertion was definied as subjective sensation of breathlessness or difficulty breathing during physical activity. Study exclusion criteria were severe comorbidities, such as heart failure (defined according to recommendations at the time [15]) with *left ventricular ejection fraction* < *50%*, chronic kidney disease, diabetes mellitus, history of lung disease (asthma, chronic obstructive pulmonary disease, pulmonary embolism) and other conditions precluding physical exercise [1]. Clinical examinations included history of reduced exercise tolerance, dyspnea, other symptoms of heart failure, and other comorbidities. Each subject underwent laboratory tests (including N-terminal prohormone of brain natriuretic peptide, NT-proBNP), echocardiography at rest (with a VIVID S6 GE Medical System, Wauwatosa, WI, USA) with a complete assessment of left ventricular systolic function (including ejection fraction and global longitudinal strain) and left ventricular diastolic function [16]. Exercise capacity was assessed with a cardiopulmonary exercise test (CPET) with the use of a Geratherm Ergostik system (Geratherm Respiratory GmbH, Germany) and a stationary ergometer exercise bike Ergoselect, following personalized ramp protocols. During CPET, hemodynamic parameters were measured continually with a PhysioFlow device (Manatec, Paris, France). The methodology has been described in detail in our previous paper [1].

The parameter of cBP was measured noninvasively via applanation tonometry with SphygmoCor XCEL (AtCorMedical, Sydney, Australia). The blood pressure cuff size was selected based on the patient’s arm circumference. Once a standard (oscillometric), automated blood pressure measurement in the right arm was recorded, the cuff was reinflated to the level of diastolic blood pressure. During the following 5 s, the brachial pressure waveform was acquired based on cuff pulsation. Subsequently, the general transfer function (GTF) of the SphygmoCor device was used to estimate the cBP waveform based on the brachial pressure waveform [16]. The cBP waveform in our study was based on such parameters as cSBP, cPP, and such parameters of arterial stiffness as AIx [17].

The difference between the blood pressure generated by the heart and the actual aortic blood pressure is referred to as augmentation pressure. The cPP parameter is defined as the difference between cSBP and central diastolic blood pressure (cDBP). The Alx is calculated as the quotient of augmentation pressure and cPP in the aorta; therefore, the Alx is independent of actual blood pressure values [18].

We measured cBP at rest prior to exercise, in 3-min intervals during exercise (started at minute 3, 6, and 9), immediately after exercise (R1), and at minute 4 of post-exercise rest (R2). It should be emphasized that the results of the measurement was obtained within about 30–45 s.

Like in other studies [14, 19] most cBP measurements (71%) were conducted at rest, at minute 3 of low-intensity exercise, and during post-exercise rest. Since measurements taken during high-intensity exercise were significantly less numerous (constituting only 33% and 17% of measurements taken at minutes 6 and 9 of exercise, respectively), they were excluded from analysis. Therefore, we considered the measurements taken at R1 to be an indirect measure of cBP at peak exercise.

### Study group

In the initial analysis 98 patients with HTN (including 52 middle-aged women; 54.5 ± 8.2 years old) were stratified by sex and evidence of dyspnea. As a result, four subgroups were formed: non-dyspneic males (*n* = 38), dyspneic males (*n* = 8), non-dyspneic females (nDFs, *n* = 27), and dyspneic females (DFs, *n* = 25) [1]. Since the dyspneic male subgroup was very small, we excluded male subjects from analysis in this study.

### Statistical analysis

Statistical analysis was performed using Statistica 12.0 (StatSoft, Inc., Tulsa, USA). Data distribution and normality were assessed by visual inspection and with the Kolmogorov–Smirnov test. Continuous variables were presented as means ± standard deviation (SD). The association between chosen variables was investigated with Spearman’s correlation coefficients.

The change in selected variables was calculated as:

Early exercise change [Ex – REST]: A difference between early exercise and resting pre-exercise absolute value = [the absolute value at the third minute of exercise] – [the baseline absolute value at rest before exercise].

Post-exercise change [R1–R2]: A difference between the first and the second post-exercise absolute value = [the absolute value of measurement at the first minute after exercise] – [the absolute value of measurement at the fourth minute after exercise].

The p-value of <0.05 was considered statistically significant.

## Results

### Baseline characteristics

No significant differences between nDFs and DFs were noted regarding baseline characteristics such as age, body mass index (BMI), N-terminal pro-B-type brain natriuretic peptide (NT-proBNP), left ventricular ejection fraction (LVEF) (Table 1). Heart failure with preserved LVEF was diagnosed in 3 (11.1%) patients of nDFs and 2 (8.0%) of DFs (non-significant difference).

**Table 1:** Baseline characteristics

	nDFs (*n* = 27)	DFs (*n* = 25)	*P* value
Variable			
Age (years), mean ± SD	56.3 ± 7.0	58.5 ± 7.3	0.268
BMI (kg/m2), mean ± SD	29.3 ± 4.5	29.4 ± 3.9	0.986
Obesity (BMI > 30 kg/m2), n (%)	12 (46.2)	11 (44.0)	0.877
Anemia (Hb<12 g/dl), *n* (%)	2 (7.7)	2 (8.0)	0.967
Nicotinism			0.955
present, *n* (%)	5 (19.2)	4 (16.0)	
in the past (>1 year), *n* (%)	5 (19.2)	5 (20.0)	
never, *n* (%)	16 (61.5)	16 (64.0)	
SpO2 (%) [REST], mean ± SD	96.8 ± 1.4	97.0 ± 1.5	0.597
Echocardiography			
LVEF (%), mean ± SD	63.9 ± 4.6	64.8 ± 4.6	0.913
LVH (IVS ≥ 12 mm), *n* (%)	8 (29.6)	5 (20.8)	0.471
NTproBNP (pg/ml), mean ± SD	103.1 ± 71.6	103.6 ± 87.7	0.984
NTproBNP > 125 (pg/ml), *n* (%)	6 (24.0)	8 (32.0)	0.528
Laboratory tests			
eGFR (ml/min/1.73 m2), mean ± SD	74.3 ± 17.5	80.6 ± 30.5	0.180
Hb (g/dl), mean ± SD	13.6 ± 1.1	13.8 ± 1.2	0.570
Glucose (mg/dl), mean ± SD	99.3 ± 17.3	98.5 ± 13.7	0.855
Total cholesterol (mg/dl), mean ± SD	212.3 ± 57.8	202.1 ± 59.3	0.532

Data presented as means ± standard deviation and absolute values (percentages)

*BMI* body mass index, *DFs* females with dyspnea, *eGFR* estimated glomerular filtration rate calculated using the Modification of Diet in Renal Disease Study (MDRD) formula, *Hb* hemoglobin, *IVS* intraventricular septum, *LVEF* left ventricular ejection fraction calculated using the *Simpson* method, *LVH* left ventricular hypertrophy, *nDFs* non-dyspneic females, *NTproBNP* N-terminal pro-B-type brain natriuretic peptide, [*REST*] value at rest, before exercise, *SpO2* pulse oxygen saturation

### Comparison between female subgroups stratified by the presence of dyspnea

Applanation tonometry results that differentiated the female subgroups stratified by dyspnea have been presented in Table 2.

**Table 2:** Applanation tonometry parameters differentiating the female subgroups stratified by dyspnea

Time point	Variable	nDFs (*n* = 27)	DFs (*n* = 25)	*P*
REST	SBP [mmHg]	137 ± 19	129 ± 12	0.093
	DBP [mmHg]	86 ± 13	80 ± 10	0.099
	cSBP [mmHg]	125 ± 15	118 ± 10	0.081
	cDBP [mmHg]	88 ± 15	80 ± 10	0.085
	cPP [mmHg]	37 ± 11	37 ± 10	0.772
	AI [%]	27 ± 15	29 ± 10	0.406
Ex	SBP [mmHg]	138 ± 20	142 ± 24	0.730
	DBP [mmHg]	89 ± 16	84 ± 12	0.334
	cSBP [mmHg]	125 ± 17	125 ± 34	0.730
	cDBP [mmHg]	91 ± 15	91 ± 16	0.743
	cPP [mmHg]	33 ± 11	41 ± 12	0.043
	AI [%]	21 ± 18	48 ± 37	0.028
Ex-REST	SBP [mmHg]	1 ± 21	11 ± 18	0.109
	DBP [mmHg]	4 ± 14	5 ± 9	1.000
	cSBP [mmHg]	0 ± 19	6 ± 30	0.214
	cDBP [mmHg]	4 ± 16	10 ± 11	0.190
	cPP [mmHg]	−4 ± 12	2 ± 12	0.105
	AI [%]	−4 ± 21	19 ± 42	0.048
R1	SBP [mmHg]	171 ± 17	168 ± 23	0.636
	DBP [mmHg]	87 ± 12	86 ± 14	0.766
	cSBP [mmHg]	143 ± 9	143 ± 17	0.886
	cDBP [mmHg]	92 ± 13	88 ± 14	0.475
	cPP [mmHg]	52 ± 13	55 ± 15	0.385
	AI [%]	6 ± 23	21 ± 16	0.048
R2	SBP [mmHg]	130 ± 19	136 ± 17	0.340
	DBP [mmHg]	78 ± 11	79 ± 10	0.641
	cSBP [mmHg]	117 ± 14	122 ± 13	0.31
	cDBP [mmHg]	80 ± 11	81 ± 11	0.846
	cPP [mmHg]	37 ± 12	41 ± 14	0.413
	AI [%]	23 ± 24	24 ± 18	0.847
R1–R2	SBP [mmHg]	40 ± 17	33 ± 23	0.240
	DBP [mmHg]	10 ± 6	7 ± 9	0.155
	cSBP [mmHg]	26 ± 11	22 ± 16	0.359
	cDBP [mmHg]	12 ± 6	8 ± 10	0.127
	cPP [mmHg]	14 ± 9	14 ± 13	0.622
	AI [%]	−18 ± 18	−6 ± 18	0.047

Data presented as means ± standard deviation and absolute values (percentages)

*AI* augmentation index, *cDBP* central diastolic blood pressure, *cSBP* central systolic blood pressure, *cPP* central pulse pressure, *DBP* central diastolic blood pressure, *DFs* females with dyspnea, *nDFs* non-dyspneic females, *SBP* systolic blood pressure, [*REST*] value at rest, before exercise, [*Ex*] value at the third minute of exercise, [*Ex* *–* *REST*] a difference between third minute of exercise and resting pre-exercise absolute value, [*R1*] value at the first minute after exercise, [*R2*] value at the fourth minute after exercise, [*R1–R2*] change in parameter between its measurements at the first and at the fourth minute after exercise

In comparison with nDFs, DFs exhibited significantly higher cPP and Alx values at minute 3 of exercise (*p* = 0.043 and *p* = 0.028, respectively) and higher AIx values immediately after exercise (*p* = 0.048). There was no difference between the subgroups in terms of cPP and AIx values at rest before exercise or at minute 4 of post-exercise rest.

Change tendency analysis showed the AIx in the nDF subgroup to decrease during exercise and reach the lowest value immediately after exercise, at which point in increased again during post-exercise rest. Conversely, the AIx value in the DF subgroup increased during the initial period of exercise, achieving decidedly higher values at minute 1 of post-exercise rest than that in the DF group. In comparison with nDFs, DFs were also characterized by a less pronounced change in Alx during post-exercise rest. Moreover, the two subgroups showed different dynamics of changes in this parameter, both during initial stages of exercise (*p* = 0.048) and at rest (*p* = 0.047). In comparison with the nDF subgroup, the DF subgroup showed a higher peak HR (145 ± 20 bpm vs. 132 ± 23 bpm, respectively).

### Correlations between central blood pressure, ergospirometric parameters, and ICG parameters

The analysis of correlations between cBP and peak HR, SV, CO, oxygen uptake (VO_2_), and oxygen pulse (O_2_Pulse) values showed inverse correlations between AIx_R1 and peak HR (*r* = −0.66, *p* < 0.001), AIx_R2 and peak HR (*r* = −0.39, *p* = 0.008), and the change in Alx (R1–R2) and peak HR (*r* = −0.45, *p* = 0.003).

There were inverse correlations between AIx_R1 and peak VO_2_(*r* = −0.41; *p* = 0.005); AIx_R1 and peak CO (*r* = −0.56; *p* < 0.0001), AIx_R2 and peak CO(*r* = −0.31; *p* = 0.040), AIx_R1-R2 and peak VO_2_ (*r* = −0.32; *p* = 0.035), and AIx_R1–R2 and peak CO (*r* = −0.40; *p* = 0.007).

## Discussion

Other authors have reported parameters of cardiopulmonary capacity to be inversely correlated with parameters of arterial stiffness at rest [14, 20–23]. Therefore, we also expected to observe differences in tonometric parameters at rest between dyspnea-based subgroups. However, there were no differences between our study subgroups in the resting values of blood pressure, cPP, or AIx. Nonetheless, we observed intergroup differences while comparing parameter change tendencies, exercise-induced values, and pressure reduction at the early post exercise phase (Table 3).

**Table 3:** Correlations between central blood pressure parameters, ergospirometric parameters, and ICG parameters during exercise testing

Time point	Variable	peak HR [bpm]	peak VO_2_ [mL/min/kg]	% pred. peak VO_2_ [%]	peak O_2_Pulse [mL/beat]	% pred. peak O_2_Pulse [%]	peak SV [mL]	peak CO [L/min]
REST	cSBP [mmHg]	0.19	0.01	−0.01	−0.31*	−0.18	0.26	0.31*
	cDBP [mmHg]	0.43*	0.08	−0.14	−0.30*	−0.47*	−0.03	0.29*
	cPP [mmHg]	−0.19	−0.07	0.12	0.01	0.21	0.25	0.00
	AI [%]	−0.10	0.08	0.14	0.15	0.20	0.04	−0.13
Ex	cSBP [mmHg]	0.11	0.11	0.20	0.00	0.09	0.28	0.12
	cDBP [mmHg]	0.44*	0.21	0.13	−0.20	−0.26	−0.18	0.12
	cPP [mmHg]	−0.28	−0.13	0.09	0.03	0.25	0.10	−0.10
	AI [%]	−0.31	0.03	0.15	0.09	0.33	0.01	−0.17
Ex-REST	cSBP [mmHg]	0.01	0.17	0.23	0.18	0.24	0.01	0.06
	cDBP [mmHg]	0.17	0.14	0.24	−0.06	0.12	−0.27	−0.17
	cPP [mmHg]	−0.15	−0.07	−0.07	0.11	0.06	0.04	−0.03
	AI [%]	−0.21	−0.02	0.07	0.07	0.18	0.03	−0.02
R1	cSBP [mmHg]	0.03	−0.20	−0.21	−0.28	−0.24	0.47*	0.31*
	cDBP [mmHg]	0.45*	0.16	−0.17	−0.35*	−0.52*	0.18	0.44*
	cPP [mmHg]	−0.40*	−0.29*	0.02	0.10	0.27	0.24	−0.13
	AI [%]	−0.67*	−0.42	−0.21*	0.15	0.28	−0.18	−0.56*
R2	cSBP [mmHg]	−0.09	−0.21	−0.13	−0.15	−0.09	0.30*	0.07
	cDBP [mmHg]	0.36*	0.19	−0.04	−0.25	−0.34*	0.04	0.32*
	cPP [mmHg]	−0.30*	−0.34*	−0.09	0.04	0.12	0.22	−0.18
	AI [%]	−0.39*	−0.28	−0.12	0.07	0.18	0.12	−0.28
R1–R2	cSBP [mmHg]	0.17	0.16	0.13	−0.04	−0.01	0.10	0.20
	cDBP [mmHg]	0.27	0.04	−0.14	−0.23	−0.34*	0.29	0.35*
	cPP [mmHg]	−0.08	0.07	0.17	0.06	0.20	−0.02	−0.01
	AI [%]	−0.45*	−0.29	−0.17	0.06	0.15	−0.23	−0.40*

*AI* augmentation index, *cDBP* central diastolic blood pressure, *CO* cardiac output, *cPP* central pulse pressure, *cSBP* central systolic blood pressure, *HR* heart rate, *O*_*2*_*Pulse* oxygen pulse, *SV* stroke volume, *VO*_*2*_ oxygen uptake, *peak* at peak exercise, *% pred. peak* percentage of predicted peak value

[*pre-Ex*] value at rest, before exercise, [*REST*] value at the third minute of exercise, [*Ex* *–* *REST*] a difference between third minute of exercise and resting pre-exercise absolute value, [*R1*] value at the first minute after exercise, [*R2*] value at the fourth minute after exercise, [*R1–R2*] change in parameter between its measurements at the first and at the fourth minute after exercise

*p < 0.05

In an earlier paper, we reported significant differences in an ICG-assessed hemodynamic response to exercise between subgroups of females stratified by dyspnea [1]. In comparison with nDFs, DFs covered a shorter distance during a 6-min walk test, had a steeper slope of the minute ventilation/carbon dioxide production (VE/VCO_2_) curve, lower peak SV values, lower peak CO, and a lower peak–rest CO change. However, there were no differences between study groups in many routine assessment results (including NT-proBNP levels, left ventricular diastolic function, and CPET parameters) [1].

In this analysis, the nDF subgroup showed a considerable decrease in AIx values during exercise (with the lowest values observed at minute 1 of post-exercise rest) and a considerable increase in AIx values during post-exercise rest. Similar changes in Alx during exercise were observed in healthy individuals [24]. Dynamic physical exercise is associated with dilation of blood vessels in active muscles, which leads to a lower vascular resistance and, consequently, a lower. Such vasodilatory response in a normally functioning vascular bed is most likely intended to optimize ventricular–arterial coupling and reduce left ventricular afterload during exercise [24].

Conversely, cBP assessment during exercise in the DF subgroup revealed a different Alx pattern, with AIx values increasing initially during exercise, and achieving also higher values at minute 1 of post-exercise rest than in the nDF subgroup. We also recorded a lower increase in AIx during post-exercise rest, which is most likely due to the lack of exercise-associated drop in AIx values, which should be reflected at the R1 time point. This may, indirectly, suggest an impaired adaptation of the cardiovascular system to undertake exercise. Other authors reported an increase in AIx at the beginning of exercise observed during static exercise [25], which was explained by an increased activation of the sympathetic nervous system. This might explain increased arterial wall tension at the beginning of exercise, a lack of artery dilation in muscles, and the consequent significantly higher AIx values at minute 3 of exercise in comparison with those in the DF subgroup. Increased sympathetic system activity further on during exercise leads to an increased chronotropic response, and the Alx is more dependent on the HR than on either inotropic effects [7, 26, 27] or pulse wave reflection [28]. As a result of increased HR during exercise, the left ventricular ejection time becomes shorter and the return of the reflected pressure wave shifts from systole to diastole, thus reducing afterload and AIx [7, 9]. Therefore, fact that high Alx values are maintained until peak exercise is even more indicative of excessive arterial stiffness and impaired hemodynamic adaptability. In nDFs we observed both lower AIx values after exercise and a greater change in AIx values during post-exercise rest, which is consistent with a greater arterial reactivity during exercise and a lower arterial stiffness in comparison with those in DFs [29].

The DF subgroup showed higher peak HR values than the nDF subgroup, which at least partly explains why DFs had lower post-exercise AIx values. The association between Alx and HR explains the inverse correlations observed between the absolute peak HR values on one hand and the absolute AIx values at minute 1 (R1) and minute 4 (R2) of post-exercise rest on the other and between peak HR and a change in AIx (R1–R2).

Correlation analyses showed that AIx values and changes in AIx during exercise were associated not only with subjectively reduced exercise tolerance but also with objectively assessed exercise capacity (peak VO_2_ and peak CO). Binder et al. [21] likewise demonstrated that a lower exercise capacity (low peak VO_2_ and low peak CO) was inversely correlated with higher Alx values at minutes 1 and 4 of post-exercise rest and with a slight increase in post-exercise Alx values. Increased arterial stiffness limits the body’s adaptability to exercise-related increases in blood volume and leads to increased left ventricular filling pressure [30, 31]. In comparison with men, women with HTN exhibit higher resting cBP, lower aortic compliance, and a clearer association between cBP and left ventricular diastolic function [3]. Increased arterial wave reflection and arterial stiffness increase systolic load, reduce CO during exercise, and—consequently—may reduce maximum VO_2_ [32]. These observations were supported by Zern et al. [14], who observed higher arterial stiffness parameters, including AIx, at rest and during exercise in women with heart failure with preserved ejection fraction in comparison with those in patients with HTN.

Reports that indicate a possibility of therapeutic interventions improving vascular response to exercise have important clinical implications. Postmenopausal women who undertook systematic exercise were observed to have an improved exercise capacity, which was associated with an improved vascular response, lower vascular resistance [33], lower arterial stiffness [34–36], and—consequently—a reduced afterload [36]. The mechanisms responsible for the phenomenon of endurance exercises possibly reducing systemic arterial stiffness include a decreased sympathetic activity and improved endothelial function [36], that is mechanisms necessary for normal cardiovascular adaptation to physical exercise [37].

### Limitations of the study

The key limitation of our study is a small sample size and the exclusion of males from analysis, with the latter dictated by the very small subgroup of males with dyspnea. Moreover, we did not assess the hormonal status of our female patients, which may affect the evaluated hemodynamic parameters. Due to a poor quality of measurements at later stages of exercise test, we were unable to assess precisely the vascular response during higher-intensity exercise.

## Conclusions

Women with HTN and dyspnea showed a different cBP response to exercise than women with HTN without dyspnea. Augmentation index and its dynamics during CPET were correlated with peak VO_2_ and CO. Evaluation of cBP parameters via applanation tonometry may proved useful in identifying hemodynamic abnormalities associated with limited exercise tolerance.

## Data Availability

The datasets generated the current study are available from the corresponding author on reasonable request.
